# Downregulation of cell surface CA125/MUC16 induces epithelial-to-mesenchymal transition and restores EGFR signalling in NIH:OVCAR3 ovarian carcinoma cells

**DOI:** 10.1038/bjc.2011.34

**Published:** 2011-02-15

**Authors:** M Comamala, M Pinard, C Thériault, I Matte, A Albert, M Boivin, J Beaudin, A Piché, C Rancourt

**Affiliations:** 1 Département de Microbiologie et Infectiologie, Faculté de Médecine et des Sciences de la Santé, Université de Sherbrooke, 3001, 12ième Avenue Nord, Sherbrooke, Quebec, Canada J1H 5N4

**Keywords:** CA125, MUC16, mucins, ovarian cancer, EMT, EGFR

## Abstract

**Background:**

Epithelial ovarian cancer (EOC) cells are prone to metastasise throughout the peritoneal cavity. The epithelial-to-mesenchymal transition (EMT) is a necessary step towards metastatic tumour progression. CA125/MUC16 mucin is a high-molecular-weight glycoprotein overexpressed in the majority of serous carcinomas, suggesting a possible role in the pathogenesis of these cancers.

**Methods:**

The role of CA125/MUC16 in EMT was investigated using single-chain antibody-mediated knockdown of cell surface CA125/MUC16 in overexpressing EOC NIH:OVCAR3 cells.

**Results:**

CA125/MUC16 knockdown was associated with morphological alterations along with decreased surface expression of epithelial markers (E-cadherin, cytokeratin-18) and increased expression of mesenchymal markers (N-cadherin, vimentin). Co-immunoprecipitation experiments revealed that CA125/MUC16 binds to E-cadherin and *β*-catenin complexes. The *in vitro* studies showed disruption of cell–cell junctions, enhanced motility, migration and invasiveness in CA125/MUC16 knockdown cells. Enhanced epidermal growth factor receptor (EGFR) activation was observed in CA125/MUC16 knockdown cells along with increased Akt and ERK1/2 phosphorylation, which are downstream effectors of EGFR, and increased MMP-2 and MMP-9 expression and activities. Epidermal growth factor receptor inhibition strongly inhibited the motility of CA125/MUC16 knockdown cells.

**Conclusions:**

Our findings suggest that CA125/MUC16 plays a role in EMT, presumably through its interaction with E-cadherin and *β*-catenin complexes and by modulating EGFR and its downstream signalling pathway in NIH:OVCAR3 cells.

Epithelial ovarian carcinomas arise from the ovarian surface epithelium (OSE) and account for the vast majority (>80%) of ovarian cancers (Ozols *et al*, 2004). Ovarian cancer is a highly metastatic disease that primarily metastasises to the serosal cavities, while dissemination through the vasculature is unusual (Naora and Montell, 2005). Current treatments for patients with metastatic ovarian carcinomas have limited efficacies with poor 5-year survival rates (Tingulstad *et al*, 2003). During the progression to a metastatic phenotype, carcinoma cells undergo morphological changes, become motile and acquire the ability to migrate and invade to establish secondary tumours at distant sites. This epithelial-to-mesenchymal transition (EMT) is characterised by coordinated molecular and cellular changes, including a reduction in cell–cell adhesion, the loss of apical–basolateral polarity, the loss of epithelial markers and the gain of mesenchymal markers (Hugo *et al*, 2007; Vergara *et al*, 2010). Epithelial-to-mesenchymal transition is an important physiological process during embryogenesis and wound healing, but also a key step in cancer metastasis (Radisky, 2005). Epithelial-to-mesenchymal transition occurs during ovarian cancer progression in response to various stimuli. A key feature of EMT is the switch from E-cadherin expression at the cell surface to N-cadherin, which promotes the interaction with stromal components (Cavallaro and Christofori, 2004).

CA125/MUC16 mucin is a high-molecular-weight glycoprotein overexpressed in the majority of serous carcinomas, the most common histological type of ovarian cancer, but is not detectable in the epithelium of normal ovaries (Kabawat *et al*, 1983; Davis *et al*, 1986; Nagata *et al*, 1991; De los Frailes *et al*, 1993; Kobayashi *et al*, 1993). These observations suggest that CA125/MUC16 may have a role in the pathogenesis of ovarian cancer. It was proposed that CA125/MUC16 influences invasiveness of benign endometriotic cell lines by acting as a chemoattractant (Gaetje *et al*, 1999). Rump *et al* (2004) reported that the interaction of CA125/MUC16 with mesothelin mediates heterotypic cell adhesion and suggested that CA125/MUC16 might contribute to the metastasis of ovarian cancer. Patankar *et al* (2005) suggested that CA125/MUC16 has potent suppression activity on natural killer cell. Structurally, CA125/MUC16 is a type I transmembrane protein consisting of an enormous *N*-terminal domain of more than 22 000 amino-acid residues that are heavily glycosylated (O’Brien *et al*, 2001, 2002) with a carbohydrate content estimated to be up to 28%, with *O*- and *N*-linked glycans (Kui *et al*, 2003). The CA125/MUC16 *C*-terminal domain is composed of an extracellular unique region that contains a potential proteolytic cleavage site, a transmembrane domain and a short cytoplasmic tail with possible phosphorylation sites (O’Brien *et al*, 2001, 2002).

This study was designed to assess the role of CA125/MUC16 in EMT. Our results show an association between CA125/MUC16 knockdown and disruption of cell–cell junctions, enhanced motility, migration and invasiveness in NIH:OVCAR3 cells. We also show that CA125/MUC16 knockdown restores EGFR activation and its downstream signalling pathway in these cells.

## Materials and methods

### Cell culture and generation of CA125/MUC16 knockdown in NIH:OVCAR3 cells

The single-chain variable fragment (scFv) was derived from the hybridoma cell line VK-8 (kindly provided by KO Lloyd, Memorial Sloan-Kettering Cancer Center, New York, NY, USA) (Lloyd *et al*, 1997), which expresses a monoclonal antibody against the extracellular domain of CA125 tumour antigen, as described previously (Boivin *et al*, 2009). The scFv DNA fragments were cloned into the pLTR.KDEL retroviral plasmid in *Sfi*I/*Not*I sites after insertion of an *Sfi*I/*Not*I containing polylinker at *Xho*I site of pLTR.KDEL, which was used to generate NIH:OVCAR3 cells stably expressing the CA125/MUC16 scFv. This vector targets the scFv to the endoplasmic reticulum where the scFv antibody is retained, thus preventing the cell surface localisation of CA125/MUC16. Two independent clones were derived (1 : 9#7 and 1 : 9#9) and were used for further studies. These clones displayed significant downregulation of cell surface CA125/MUC16 (Boivin *et al*, 2009). A control subline was also derived, which express an scFv that does not bind CA125/MUC16. The human ovarian cancer cell line NIH:OVCAR3 was obtained from the American Type Culture Collection (Rockville, MD, USA) and grown in RPMI 1640 (Wisent, St-Bruno, QC, Canada) supplemented with 20% FBS (Wisent), 2 mM L-glutamine (Wisent), 100 U ml^−1^ penicillin, 100 *μ*g ml^−1^ streptomycin and 10 *μ*g ml^−1^ insulin (Wisent). Cells were maintained at 37°C in humidified 5% CO_2_ incubators. NIH:OVCAR3 subclones were maintained in media with blasticidin 0.5–1 *μ*g ml^−1^ (Invitrogen, Burlington, ON, Canada). Fragments of the OSE were scraped from the ovarian surface following a surgery procedure for non-malignant gynaecological conditions. The fragments were incubated in 4 ml of OSE medium (Wisent) supplemented with 10% FBS and 10 nM *β*-oestradiol in a T75 flask. Cells were washed with 2 ml of OSE medium. After 2 days, fresh OSE medium was added and the medium was changed once in week after.

### Reagents

Anti-E-cadherin antibody (clone 67A4) was from Chemicon International Inc. (Billerica, MA, USA). Anti-claudin-7 (clone 5D10F3) and anti-cytokeratin 8–18 (clone zym5.2) antibodies were from Zymed Laboratories Inc. (San Francisco, CA, USA). Anti-tubulin antibody was from Sigma (Oakville, ON, Canada), and anti-vimentin, anti-ERK1/2, anti-EGFR and anti-N-cadherin antibodies were from Santa Cruz Biotechnology Inc. (Santa Cruz, CA, USA). Antibodies for Akt, ERK1/2 and EGFR were from Cell Signaling (Pickering, ON, Canada). Antibodies for phospho-Akt (Ser-473), phospho-EGFR (Y-1068) and phospho-ERK1/2 were from Invitrogen. Anti-CA125 (M11) was from Dako (Burlington, ON, Canada). Epidermal growth factor receptor inhibitors AG1478 and PD153035 were obtained from Calbiochem Corp. (La Jolla, CA, USA). Epidermal growth factor was purchased from R&D Systems (Minneapolis, MN, USA). Hepatocyte growth factor and TGF-*β* were from PeproTech (Rocky Hill, NS, USA).

### Immunoblot analysis

For the detection of phosphoproteins, cells were lysed in Nonidet P-40 isotonic lysis buffer (283 mM KCl, 10 mM MgCl_2_, 50 mM HEPES, pH 7.2, 4 mM EGTA, 0.5% NP-40, 10 mM sodium fluoride, 100 *μ*M sodium pyrophosphate, 400 *μ*M sodium orthovanadate with freshly added protease inhibitors (1 *μ*g ml^−1^ 4-(2-aminoethyl) benzenesulphonyl fluoride hydrochloride, 20 *μ*g ml^−1^ aprotinin, 0.7 *μ*g ml^−1^ pepstatin and 1 *μ*g ml^−1^ leupeptin) (Sigma)) and proteins were quantified by Bradford assay (Bio-Rad, Hercules, CA, USA). For the detection of other proteins, cells were lysed in lysis buffer containing Triton X-100 (1%), 20 mM Tris (pH 7.5), 150 mM NaCl, 1 mM EDTA and 1 mM EGTA. Control and knockdown cell lysates (equal amounts of proteins) were submitted to SDS–PAGE electrophoresis and transferred onto PVDF membrane (Roche, Laval, QC, Canada). The membranes were probed with N-cadherin, vimentin, phospho-EGFR, EGFR, phospho-Akt, Akt, phospho-ERK1/2, ERK1/2 or tubulin antibodies. Anti-mouse- and anti-rabbit-conjugated HPR secondary antibodies were from Cell Signaling. The immunoblots were developed by chemiluminescence using the ECL plus system according to the manufacturer's instruction (GE Healthcare, Baie d’Urfé, QC, Canada).

### Immunofluorescence

Ovarian surface epithelium cells and stable NIH:OVCAR3 derivative sublines expressing the various scFvs were grown on glass slides until a 50–70% confluence was reached. Glass slides were then washed in cold PBS and cells fixed in 3.7% formaldehyde for 15 min on ice. Depending on the experiment, cells were permeabilised with PBS containing 0.2% Triton X-100 for 20 min at room temperature. Slides were rinsed twice in PBS and blocked in PBS/2% BSA at room temperature for 1 h and then incubated with primary antibodies in blocking buffer at room temperature for 1 h. Slides were washed two times in cold PBS, incubated for 1 h at room temperature with Alexa Fluor 594 (red) or Alexa Fluor 488 (green) F(ab′)_2_ fragment of goat anti-mouse or goat anti-rabbit IgG (Invitrogen). In some experiments, F-actin was visualised using phalloidin–rhodamine staining (Sigma). After washing, slides were incubated for 2 min in 4′, 6′-diamidino-2-phenylindole (DAPI) to visualise the nuclei, washed again in PBS and mounted for visualisation by fluorescence microscopy with an Olympus IX70 (Olympus, Hamburg, Germany).

### Spheroid formation assay

Spheroid formation assays were performed with the parental cell line, Ctrl scFv cells and CA125/MUC16 knockdown cells. A 25 *μ*l drop of medium containing 5 × 10^3^ cells was pipetted onto the inner surface of a Petri dish lid. The lid was then placed on the Petri dish so that the drops were hanging from the lid with the cells suspended within them. To eliminate evaporation, 10 ml serum-free culture medium was placed in the bottom of the Petri dish. After overnight incubation at 37°C, the lid of the Petri dish was inverted and photographed using an inverted tissue culture microscope at × 40 magnification.

### Wound healing assay

Cells were seeded onto six-well plates and when they reached 100% confluence, a scratch was made across the cell monolayer. Cells were gently washed with PBS and new media containing hydroxyurea 30 mM (Sigma, Steinheim, Germany) were added to block cell division. Cells were incubated for 24 h and photographed using an inverted tissue culture microscope at × 100 magnification.

### Motility and invasion assay

For motility assay, 5 × 10^3^ cells were seeded in the top chamber of monolayer-coated polyethylene terephthalate membranes (six-well insert, pore size of 8 *μ*M) (Becton Dickinson, Mississauga, ON, Canada). The cells were incubated for 16–20 h, and cells that did not migrate through the membrane were removed by scraping with a cotton swab. Cells that migrated through the membrane were fixed with methanol and stained with crystal violet 1% and cells in 10 random fields were counted at × 100 magnification. For invasion assay, 5 × 10^3^ cells were seeded on Matrigel-coated membrane inserts (Becton Dickinson). The bottom chamber contained 0.75 ml RPMI supplemented with 10% foetal bovine serum as a chemoattractant. Cells were incubated for 24 h, the cells remaining inside the insert were removed with a cotton swab and cells that had penetrated the Matrigel to invade to the lower surface of the membrane were fixed in methanol and stained with crystal violet 1%. After air drying the membrane, the cells were counted at × 100 magnification in 10 random fields of view under a microscope. Three independent experiments were performed in each case.

### Zymography

Matrix metalloproteinase-2 and MMP-9 activity in the conditioned medium of parental NIH:OVCAR3, Ctrl scFv and CA125/MUC16 knockdown cells were analysed using 10% SDS gelatin zymography. Cells were seeded in six-well plates and grown at a density of 80% and cells were incubated for an additional 48 h in medium without FBS. Electrophoresis of conditioned medium was performed in acrylamide gel containing 2 *μ*g ml^−1^ of gelatin. After running, the gel was incubated for 3 h in 0.25% Triton X-100 and the renaturing buffer was replaced with developing buffer (Tris-HCl 50 mM, NaCl 0.2 M, CaCl_2_ 5 mM, Brij 35 0.02%) for 30 min at room temperature. It was then replaced with fresh developing buffer and the gel was incubated at 37°C for 16 h. The gel was stained with Coomassie Blue R-250 to visualise protease activity. Blood was used as a positive control for the activation of these metalloproteinases.

### Electronic microscopy

Cells were washed with cacodylate 0.1 M (pH 7.4) and fixed in 1.4% glutaraldehyde at room temperature for 30 min, and then in 2.5% glutaraldehyde overnight at 4°C. Cells were contrasted with 1% osmium tetroxide and 1% uranyl acetate. Cells were dehydrated in successive washes of 70, 90 and 100% ethanol before embedding in EPON 3 X. Blocks were frozen onto mounting pins and 70–80 nm sections were collected. Images were captured with a transmission electron microscope (Hitachi H-7500) using AMT Software (Boston, MA, USA).

### Statistical analysis

Motility and invasion assay results were compared between the parental cell line and Ctrl scFv and 1 : 9#7 and 1 : 9#9 knockdown cells by the two-tailed Student's *t*-test. A *P*-value of ⩽0.05 was considered statistically significant.

## Results

### Morphological changes induced by the downregulation of CA125/MUC16

**Figure 1:**
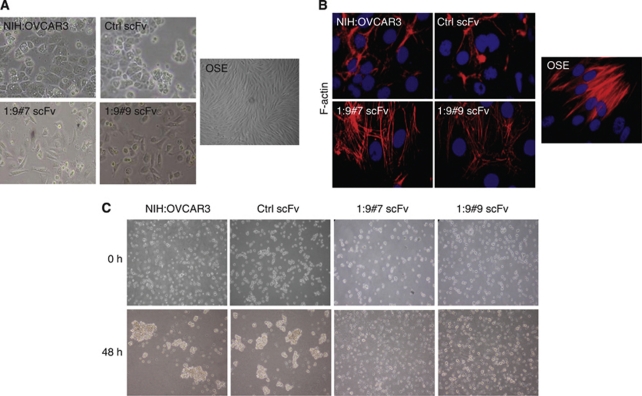
CA125/MUC16 knockdown alters cell morphology and spheroid formation of NIH:OVCAR3 cells. (**A**) The morphology of control, CA125/MUC16 knockdown in NIH:OVCAR3 cells and OSE cells by phase-contrast microscopy ( × 200 magnification). (**B**) Immunofluorescence analysis of control, CA125/MUC16 knockdown cells and OSE cells ( × 1000 magnification). The panels show phalloidin–rhodamine staining of actin-cytoskeleton changes induced by CA125/MUC16 knockdown and DAPI for nuclear staining. (**C**) Control and knockdown cells were seeded in suspension for this aggregation assay. Control cells showed bigger and tighter cell aggregates in contrast to knockdown cells ( × 40 magnification).

We have chosen the widely used NIH:OVCAR3 epithelial ovarian carcinoma cell line for our studies because, like most serous ovarian carcinomas, these cells overexpress CA125/MUC16 at the their surface and they show a complete epithelial phenotype (Hamilton *et al*, 1983; Yin and Lloyd, 2001; Rump *et al*, 2004). In addition, NIH:OVCAR3 cells are non-invasive in Boyden chambers, poorly anchorage-independent, non-motile on plastic and do not invade in collagen gels (Hamilton *et al*, 1987). These properties make NIH:OVCAR3 cells ideally suitable to study EMT. To overcome the difficulties associated with silencing the large size of CA125/MUC16 RNA, we used a system where the cell surface expression of CA125/MUC16 in NIH:OVCAR3 cells was downregulated by the expression of an endoplasmic reticulum-localised CA125/MUC16 scFv to study its role in EMT (Gubbels *et al*, 2006; Boivin *et al*, 2009). The expression of cell surface CA125/MUC16 was reduced by 90% in two independent NIH:OVCAR3 CA125/MUC16 scFv-expressing subclones (1 : 9#7 and 1 : 9#9 scFvs), whereas control NIH:OVCAR3 expressing a control (Ctrl) scFv did not show alteration of CA125/MUC16 expression compared with the parental NIH:OVCAR3 (Boivin *et al*, 2009). We noticed that CA125/MUC16 knockdown in NIH:OVCAR3 cells display significant changes in morphological features compared with controls. Control cells (parental NIH:OVCAR3 and Ctrl scFv cells) formed cobblestone-like monolayer, round boundary and cell–cell junctions and adhesion between neighbouring cells (Figure 1A). In contrast, knockdown cells have a longer and fibroblast-like shape more characteristic of mesenchymal cells and display scattering of the cells. These changes were compared with OSE cells that are CA125/MUC16 negative and display mesenchymal features as indicated by their fibroblast-like shape (Figure 1A). The distribution of filamentous actin was analysed by phalloidin staining and clearly revealed alterations in the actin cytoskeleton from the predominance of cortical actin (control cells) into actin stress fibres throughout the cells for CA125/MUC16 knockdown and OSE cells (Figure 1B). Re-organisation of filamentous actin is characteristic of a cell-spreading response (Cunningham *et al*, 1992). These observations suggest that the CA125/MUC16 knockdown induces a phenotypic switch in carcinoma cells that resemble OSE cells.

Next, the NIH:OVCAR3 sublines were plated under anchorage-independent conditions and assessed, 48 h later, for the formation of cell spheroids that were visualised by phase-contrast microscopy. As shown in Figure 1C, the Ctrl scFv-expressing cells and parental NIH:OVCAR3 cells formed large floating spheroids, whereas CA125/MUC16 knockdown cells appeared as single isolated cells after 48 h. Increasing the incubation time to 72 h did not allow the CA125/MUC16 knockdown cells to form cell aggregates (data not shown). These findings show that the downregulation of CA125/MUC16 cell surface expression prevents homotypic cell aggregation and is associated with morphological changes suggestive of EMT.

### Expression of mesenchymal markers in CA125/MUC16 knockdown cells

**Figure 2:**
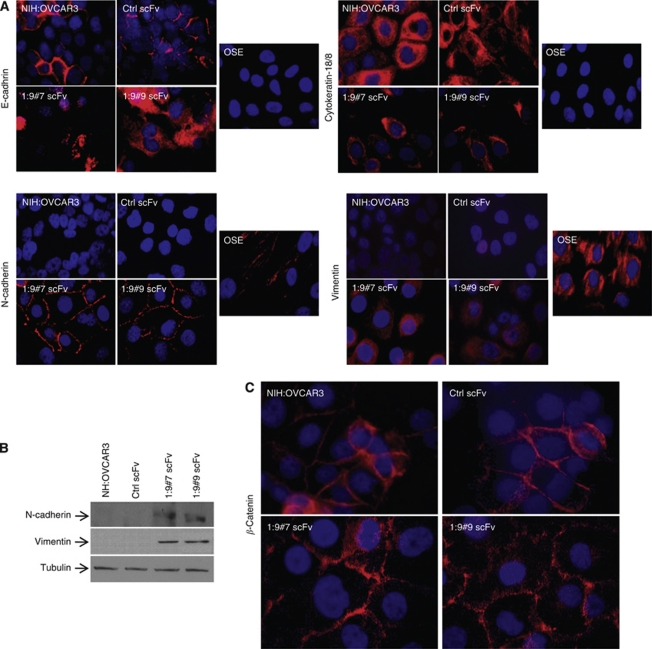
CA125/MUC16 knockdown alters expression of epithelial and mesenchymal markers. (**A**) Immunofluorescence analysis showing a decreased or absence of cell surface expression of E-cadherin and cytokeratin-18 and increased expression of N-cadherin and vimentin in CA125/MUC16 knockdown and OSE cells ( × 1000 magnification). (**B**) Immunoblot analysis of N-cadherin and vimentin expression in control and knockdown cells. (**C**) Immunofluorescence analysis of *β*-catenin expression in control and CA125/MUC16 knockdown cells.

Epithelial-to-mesenchymal transition is a process characterised by the loss of epithelial markers such as E-cadherin and cytokeratin-18 and gain of mesenchymal markers such as N-cadherin and vimentin (Thiery, 2003; Ahmed *et al*, 2007; Vergara *et al*, 2010). During progression, carcinoma cells with increased invasiveness and metastatic potential usually acquire mesenchymal markers and show a reduction or absence of E-cadherin expression (Hugo *et al*, 2007). However, in contrast to most adenocarcinomas, advanced and poorly differentiated ovarian tumours continue to express E-cadherin (Ahmed *et al*, 2007). This contrasts with OSE cells, which do not express E-cadherin (Auersperg *et al*, 2001). Immunofluorescence microscopy analysis showed that E-cadherin was detected at the contacts between NIH:OVCAR3 control cells, whereas there was a loss of E-cadherin staining at the cell surface and an internalisation of E-cadherin in CA125/MUC16 knockdown cells (Figure 2A). As expected, OSE did not express E-cadherin. Knockdown cells behave very much like OSE cells; they displayed decreased expression of cytokeratin-18, whereas N-cadherin and vimentin expression were increased compared with NIH:OVCAR3- and Ctrl scFv-transfected cells (Figure 2A). Western blot analysis confirmed the increased expression of N-cadherin and vimentin in CA125/MUC16 knockdown cells (Figure 2B). The cytoplasmic domain of E-cadherin has been shown to bind to *β*-catenin (Sasaki *et al*, 2000). *β*-Catenin staining was observed to localise at the contacts of cells in a manner similar to E-cadherin in NIH:OVCAR3- and Ctrl scFv-transfected cells, but partially re-localised in the cytoplasm in CA125/MUC16 knockdown cells (Figure 2C). Altogether, these data show that CA125/MUC16 knockdown induces a re-distribution of E-cadherin and *β*-catenin epithelial markers throughout the cytoplasm and increased expression of mesenchymal markers, such as N-cadherin and vimentin, consistent with an EMT. The data also show that OVCAR3 knockdown cells acquired a phenotype that was very similar to that of CA125-negative OSE cells.

### CA125/MUC16 binds to E-cadherin and *β*-catenin complexes

**Figure 3:**
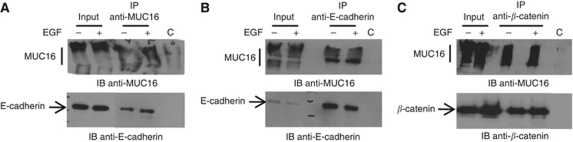
Association of CA125/MUC16 with E-cadherin and *β*-catenin. Lysate from adherent NIH:OVCAR3 cells were immunoprecipitated with anti-CA125/MUC16 (M11) antibody (**A**), anti-E-cadherin antibody (**B**) or anti-*β*-catenin antibody (**C**) in the presence or absence of EGF. Lysates were also immunoprecipitated with a control IgG (c). The immunoprecipitates were analysed for reactivity with CA125/MUC16, E-cadherin and *β*-catenin. Lysates were directly analysed by immunoblotting as controls (input).

As MUC1 has been previously shown to bind *β*-catenin (Yamamoto *et al*, 1997), and as E-cadherin and *β*-catenin expressions were altered by the knock down of CA125/MUC16, we assessed whether these proteins associate with CA125/MUC16 in the presence or absence of EGF because EGF may affect E-cadherin expression (Lu *et al*, 2003). Co-immunoprecipitation experiments confirmed the association of CA125/MUC16 with E-cadherin complexes in NIH:OVCAR3 cells. As shown in Figure 3A, antibodies to CA125/MUC16 immunoprecipitated CA125/MUC16 from the lysates of NIH:OVCAR3 cells. More importantly, E-cadherin was co-immunoprecipitated with CA125/MUC16 in these cells with or without EGF treatment. In reciprocal experiments, antibodies to E-cadherin immunoprecipitated E-cadherin and co-immunoprecipitated CA125/MUC16 from the lysate of NIH:OVCAR3 cells, suggesting that CA125/MUC16 forms complexes with E-cadherin (Figure 3B). In a similar set of experiments, we found that antibodies to *β*-catenin co-immunoprecipitated CA125/MUC16 (Figure 3C). These findings indicate that CA125/MUC16 associates with E-cadherin and *β*-catenin complexes in NIH:OVCAR3 cells.

### The knock down of CA125/MUC16 enhances migration and disruption of cell–cell junctions of NIH:OVCAR3 cells *in vitro*

**Figure 4:**
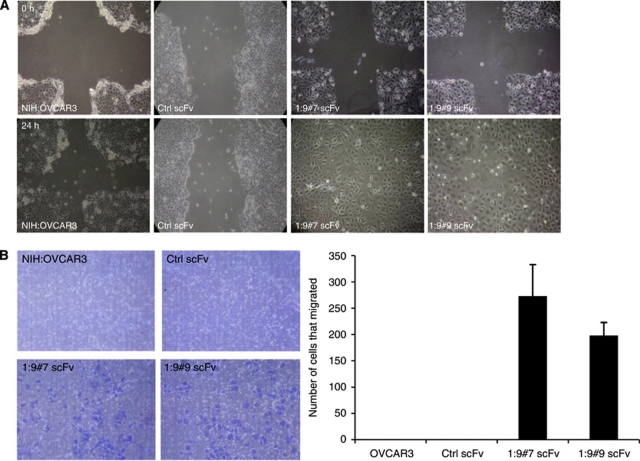
CA125/MUC16 knockdown enhances cell motility. (**A**) Wound healing assay was performed as described in Materials and Methods to assess the impact of CA125/MUC16 knockdown of cell motility ( × 100 magnification). Upper panels show the initial wound and lower panels show the same wound 24 h later. (**B**) Boyden's chamber motility assay for control and knockdown cells. Cell motility was significantly enhanced (*P*<0.001) in CA125/MUC16 knockdown cells ( × 100 magnification).

Epithelial-to-mesenchymal transition is characterised by increased motility and invasiveness (Hugo *et al*, 2007). To determine whether CA125/MUC16 knockdown cells have acquired the ability to migrate, we performed a wound healing assay in which the motility of cells located at the edge of the wound was evaluated on the basis of their ability to colonise the wounded area. In the presence of serum (10% FBS) and hydroxyurea 30 mM (to block cell division), wound repair was observed only with the CA125/MUC16 knockdown cells at 24 h (Figure 4A). These results were confirmed using a transwell chamber assay. As shown in Figure 4B, a substantial number of CA125/MUC16 knockdown cells migrated through the pores, whereas control cells did not migrate at all.

**Figure 5:**
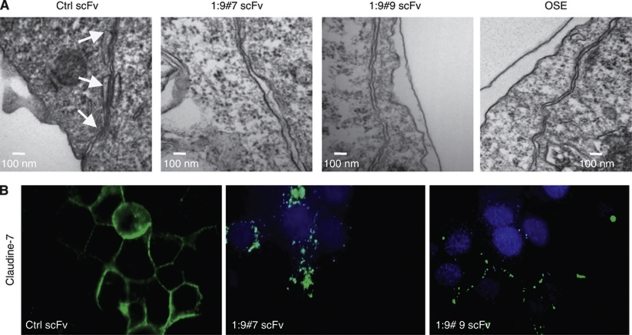
CA125/MUC16 knockdown disrupts cell–cell junctions. (**A**) Assessment of cell–cell junctions in Ctrl scFv and CA125/MUC16 knockdown cells and OSE cells by electronic microscopy. Arrows indicate the presence of three desmosome spots in Ctrl scFv cells and the lack of such desmosomes in knockdown cells (scale bar – 100 nm). (**B**) Immunofluorescence analysis of claudin-7 expression, a protein involved in tight junctions, in Ctrl scFv and knockdown cells showing the disruption of the protein at the cell surface of knockdown cells ( × 1000 magnification).

Epithelial-to-mesenchymal transition usually involves the disruption of tight junctions, adherens junctions and desmosomes, which contribute to the separation into individual cells (Sundfeldt, 2003). Because of the morphological changes and the absence of spheroid formation in suspension observed in CA125/MUC16 knockdown cells (Figure 1), and their enhanced potential to migrate (Figure 4), we evaluated cell junctions by electronic microscopy in controls and knockdown NIH:OVCAR3 cells. Figure 5A shows a representative electron micrograph of three spot desmosomes between two Ctrl scFv-expressing NIH:OVCAR3 cells forming adhering junctions. In contrast, OSE and CA125/MUC16 knockdown cells lack such adhering junctions, resulting in larger intercellular space. Consistent with these results, the staining pattern for claudin-7 proteins, which are involved in tight junctions, were altered in CA125/MUC16 knockdown cells as compared with control cells (Figure 5B). Altogether, these data show that CA125/MUC16 knockdown in NIH:OVCAR3 cells disrupts cell–cell junctions and promotes cell migration.

### The knock down of CA125/MUC16 induces an invasive phenotype

**Figure 6:**
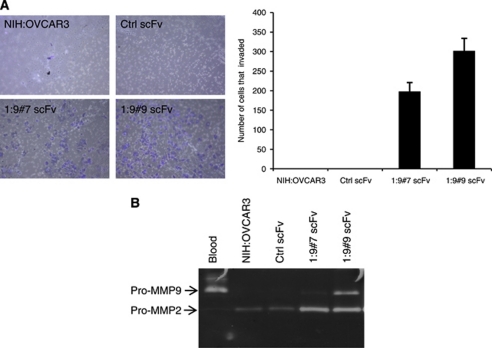
CA125/MUC16 knockdown increases the invasive phenotype. (**A**) Matrigel-coated Boyden's chamber were used to assess the effect of CA125/MUC16 knockdown on invasive property of NIH:OVCAR3. CA125/MUC16 knockdown cells exhibited a significant increase in invasiveness compared with parental NIH:OVCAR3 and Ctrl scFv cells (*P*<0.001; × 100 magnification). (**B**) Secreted MMP-2 and MMP-9 activities in cell-free conditioned medium is increased in CA125/MUC16 knockdown cells compared with control NIH:OVCAR3 and Ctrl scFv cells. Blood was used as a positive control of MMP-2 and MMP-9 activity.

The invasive ability of control and CA125/MUC16 knockdown cells was examined by the transwell chamber assay containing an extracellular matrix layer (Matrigel). As shown in Figure 6A, CA125/MUC16 knockdown cells exhibited a significant increase in invasion compared with control scFv-expressing cells and parental NIH:OVCAR3 cells, which were unable to invade. Because the downregulation of CA125/MUC16 in NIH:OVCAR3 cells was associated with an invasive phenotype, we next investigated whether knockdown cells show an induction of MMP activity, a key event in the disruption of membranes for the invasion of tumour cells (Suyama *et al*, 2002). Analysis of MMP levels in a gelatin zymographic assay showed that the activity of pro-MMP2 and pro-MMP9 was substantially enhanced in knockdown cells consistent with their greater capacity of invasion (Figure 6B). These results indicate that the knock down of CA125/MUC16 promotes the ability of NIH:OVCAR3 cells to migrate and invade as part of their EMT acquired phenotype.

### The knock down of CA125/MUC16 EGFR restores its downstream signalling

**Figure 7:**
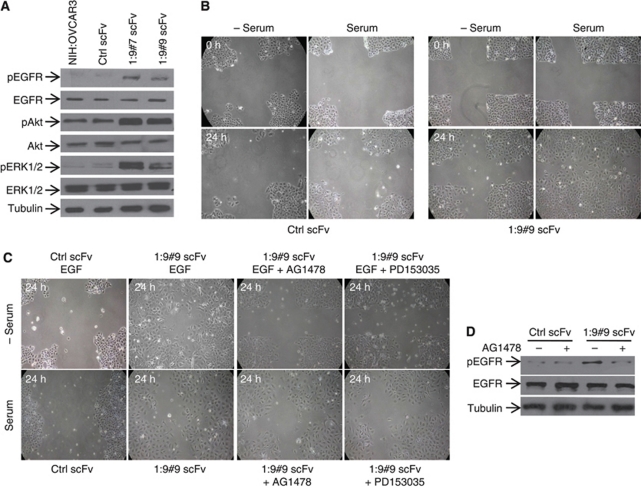
CA125/MUC16 knockdown activates EGFR and its inhibition reduces the motility of these cells. (**A**) Western blot analysis showed activation of EGFR and its downstream targets Akt and ERK1/2 in CA125/MUC16 knockdown cells as compared with NIH:OVCAR3 and Ctrl scFv cells. (**B**) Wound healing assay in Ctrl scFv and CA125/MUC16 knockdown 1 : 9#9 cells in the presence or absence of serum (20% FBS). The migration of CA125/MUC16 knockdown cells was dependent on the presence of serum ( × 100 magnification). (**C**) Wound healing assay in Ctrl scFv and CA125/MUC16 knockdown 1 : 9#9 cells treated with serum, EGF (10 ng ml^−1^) and EGF inhibitors AG1478 (4 *μ*M) or PD153035 (5 *μ*M). EGF had an effect similar to serum on the motility of CA125/MUC16 cells, but serum failed to stimulate migration in Ctrl scFv cells. The presence of EGF inhibitor strongly decreased serum- or EGF-induced motility in knockdown cells ( × 100 magnification). (**D**) Western blot analysis of EGFR activation in Ctrl scFv and CA125/MUC16 knockdown 1 : 9#9 cells in the presence or absence of AG1478. AG1478 completely abrogated serum-mediated EGFR activation in knockdown cells.

We analysed the mechanism of CA135/MUC16-induced EMT in NIH:OVCAR3 cells. We observed an increase in the phosphorylation of EGFR (Y1068) in the CA125/MUC16 knockdown cells, although the levels of total EGFR remained unchanged (Figure 7A). We also determined the expression and phosphorylation of Akt and ERK1/2, which are downstream signalling molecules induced upon EGFR activation. CA125/MUC16 knockdown-induced activation of EGFR led to the activation of Akt and ERK1/2 as shown in Figure 7A. These data suggest that the knock down of CA125/MUC16 modulates the phosphorylation of EGFR and activation of its downstream targets to promote EMT.

Under physiological conditions, EMT of OSE cells is induced by growth factors (Ahmed *et al*, 2006). For example, in primary cultures of OSE cells, the addition of EGF induced morphological changes consistent with EMT and enhanced motility and MMP-2/-9 activity (Ahmed *et al*, 2006). Furthermore, in prostate cancer cells, EGF treatment can promote tumour cell motility and invasion (Lu *et al*, 2003). Finally, the knock down of CA125/MUC16 activates EGFR as described above. We thus examined the ability of serum to induce a motile phenotype in the controls and CA125/MUC16 knockdown cells using the wounding assay. As shown in Figure 7B, when cells were incubated in the absence of serum, no wound repair was observed in either control scFv or 1 : 9#9 scFv-expressing cells. Although the presence of serum (10% FBS) also failed to promote wound repair in control scFv cells, it strongly enhanced the motility of 1 : 9#9 scFv-expressing cells (Figure 7B). In addition to EGF, other growth factors present in the serum, such as TFG-*β* and HGF, have been involved in promoting cell motility (Kalluri and Neilson, 2003). EGF treatment (100 ng ml^−1^), in the absence of serum, promoted wound repair of 1 : 9#9 scFv-expressing cells within 24 h, whereas the motility of ctrl scFv-expressing cells was not enhanced (Figure 7B). Similar experiments carried out with HGF (20 ng ml^−1^) or TGF-*β* (10 ng ml^−1^) in the absence of serum failed to promote wound repair of 1 : 9#9 scFv-expressing cells (data not shown). Treatment of 1 : 9#9 scFv-expressing cells with specific EGFR inhibitors AG1478 and PD153035 resulted in the inhibition of EGF- and serum-induced cell motility (Figure 7C). The stimulation of cell motility by the serum in 1 : 9#9 scFv-expressing cells correlated with EGFR activation and serum-induced EGFR activation was blocked by AG1478 (Figure 7D). The results show that EGF is an important component of serum that stimulates cell motility in CA125/MUC16 knockdown cells.

## Discussion

Since it was first described in 1981 (Bast *et al*, 1981, 1983), the biological function of ovarian cancer tumour antigen CA125/MUC16 has remained mostly unknown. Only recently, convincing evidence showed that it binds to mesothelin (Rump *et al*, 2004) and galectin-1 (Seelenmeyer *et al*, 2003). As these proteins are involved in cell adhesion, one could speculate that CA125MUC16 is involved in cell adhesion and perhaps participates to the metastatic process of ovarian cancer. Altered expression of mucins such as MUC1 and MUC4 has been shown to regulate various processes such as tumour cell growth, cell adhesion, motility and tumorigenicity (Wesseling *et al*, 1995; Satoh *et al*, 2000; Komatsu *et al*, 2001; McDermott *et al*, 2001; Singh *et al*, 2004; Ponnusamy *et al*, 2010). By analogy with these mucins, it may be expected that CA125MUC16 plays similar functions in ovarian cancer. However, direct experimental evidences until now were lacking to support such roles. In this study, we provide evidence that CA125/MUC16 affects cellular behaviours in NIH:OVCAR3 cells, including changes in morphology and motility. At the molecular level, the knock down of CA125/MUC16 induces significant alteration of EMT markers. In addition, CA125/MUC16 knockdown activates EGFR and its downstream targets Akt and ERK1/2 to promote cell motility. Furthermore, the knock down of CA125/MUC16 promoted cell migration and invasion of NIH:OVCAR3 cells. To the best of our knowledge, this is the first report showing the role of CA125/MUC16 in EMT.

Ovarian cancer cells are prone to metastasise throughout the peritoneal cavity. Metastasis is a complex process involving changes in cell–extracellular matrix and cell–cell junctions. Epithelial-to-mesenchymal transition is a necessary step towards metastatic tumour progression during detachment of tumour cells from the primary tumour site and attachment to metastatic sites. Epithelial-to-mesenchymal transition results in enhanced cell motility and invasion. Few studies have examined the factors that promote EMT of epithelial ovarian cancer (EOC) cells (Rosano *et al*, 2005; Theriault *et al*, 2007; Pon *et al*, 2008). Here we show that CA125/MUC16 cell surface knockdown induces EMT in NIH:OVCAR3 cells. During progression, EOC cells tend to detach from each other and disseminate within the peritoneal cavity. Our data suggest that CA125/MUC16 could be involved in this process. Interestingly, we showed that CA125/MUC16 knockdown induces an intracellular relocalisation of E-cadherin (Figure 2A). The re-distribution of E-cadherin in the cytoplasm, the decreased expression of cytokeratin-18 and the gain of mesenchymal markers N-cadherin and vimentin are the hallmarks of EMT (Thiery, 2003). Ovarian carcinoma are unique in that epithelial differentiation becomes more, rather than less, prominent as the tumour develop. This increased epithelial differentiation is associated with an increase in E-cadherin expression. However, despite the fact that ovarian tumours express E-cadherin, ovarian carcinoma metastases often show the absence of E-cadherin expression, suggesting that the loss of E-cadherin is associated with the ability of ovarian tumour to metastasise (Sundfeldt, 2003). Similarly, the loss of tissue CA125/MUC16 expression is associated with late-stage EOC tumour (Hogdall *et al*, 2007). It is possible that binding of CA125/MUC16 to E-cadherin complexes results in the surface localisation of E-cadherin, which mediates cell contact and suppression of cell invasion. Conversely, in the absence of CA125/MUC16, E-cadherin relocalises in the cytoplasm, which abolishes its ability to promote cell contact formation. This is supported by the observations that CA125/MUC16 associates with E-cadherin complexes (Figure 3), the fact that knock down of CA125/MUC16 induces E-cadherin relocalisation in the cytoplasm (Figure 2A) and that E-cadherin relocalisation in knockdown NIH:OVCAR3 cells is associated with increased motility (Figure 4). Our data also show that CA125/MUC16 forms complexes with *β*-catenin. The cytoplasmic domain of E-cadherin binds to *β*-catenin, which forms complexes with *α*-catenin (Ozawa *et al*, 1990), actin (Adams *et al*, 1996), p120 (Staddon *et al*, 1995), EGFR (Hoschuetzky *et al*, 1994) and other proteins. It is possible that by forming a complex with E-cadherin and/or *β*-catenin, CA125/MUC16 could re-distribute EGFR and consequently modulates its signalling pathway.

CA125/MUC16 tandem repeats were identified as interacting with mesothelin and that this interaction mediates binding of cells expressing CA125/MUC16 tandem repeats to mesothelial cells or mesothelin-expressing cells. CA125/MUC16 may be involved in metastasis of ovarian cancers by mediating cell attachment to mesothelial cells of the peritoneal lining (Rump *et al*, 2004). The results presented herein are consistent with this report and the suggestion that CA125/MUC16 modulates cell adhesion properties. However, based on the approach used to downregulate CA125/MUC16, it is impossible to assign a specific role to the large *N*-terminal domain of CA125/MUC16 in regulating the EMT process.

CA125/MUC16 is a serum marker that is elevated in more than 80% of EOC patients (Canney *et al*, 1984; Vergote *et al*, 1987). In a recent study, the loss of tissue CA125/MUC16 was associated with late-stage primary EOC tumour. Furthermore, the loss of CA125/MUC16 expression significantly correlated with poor survival (Hogdall *et al*, 2007). The demonstration that downregulation of cell surface CA125/MUC16 in NIH:OVCAR3 cells results in increased motility and invasion is therefore consistent with these findings. The loss of cell surface CA125/MUC16 in late-stage tumours could enhance their ability to metastasise. Although little is known about the signalling pathways regulated by CA125/MUC16, its downregulation was associated with the upregulation of N-cadherin and vimentin, two well-established mesenchymal markers. Whether CA125/MUC16 directly regulates the expression of these mesenchymal markers remains to be determined. However, a recent study showed that MUC4 modulates N-cadherin expression in an FAK-dependent manner in a pancreatic cell line (Shintani *et al*, 2008). Furthermore, Huang *et al* (2005) showed that MUC1 cytoplasmic domain binds *β*-catenin and can therefore compete with E-cadherin for binding to *β*-catenin.

The OSE is a major target tissue for ovarian carcinoma formation. With each ovulation, OSE cells become highly migratory so that they can fill the large wound that is generated during oocyte release. This phenotypic switch to a mesenchymal, non-cohesive migratory phenotype also occurs when normal human OSE cells are explanted into monolayer culture, which likely reflects a primitive differentiation state that may be facilitated by an absence of E-cadherin and/or CA125/MUC16 in these cells (Auersperg *et al*, 2001). In contrast, well-differentiated EOC are non-migratory and they express CA125/MUC16 and E-cadherin at their cell surface. Therefore, cell surface expression of E-cadherin and CA125/MUC16 may be functionally important during ovarian carcinoma formation. Indeed, ectopic expression of E-cadherin in OSE cells induces a phenotypic switch from mesenchymal-to-epithelial-like properties (Wu *et al*, 2008). Furthermore, our data showed that CA125/MUC16 knockdown in OVCAR3 cells, which is associated with the loss of cell surface E-cadherin expression, induced a switch from epithelial-to-mesenchymal features. Thus, CA125/MUC16 knockdown in OVCAR3 cells behave like CA125/MUC16-negative OSE cells with regard to EMT markers.

On the basis of the previous finding of EGF-induced EMT in human OSE (Ahmed *et al*, 2006), we characterised the mechanism underlying CA125/MUC16-induced EMT by showing that CA125/MUC16 knockdown activates EGFR and its downstream signalling in NIH:OVCAR3 cells. We observed an increase in the activation of Akt, ERK1/2 and MMP-2 and MMP-9 in CA125/MUC16 knockdown cells. Activation of the MAPK-ERK pathway has been shown to upregulate MMP-9 and enhanced cell migration (Suyama *et al*, 2002). In NIH:OVCAR3 cells, the increased phosphorylation of ERK1/2 induced by the knockdown of CA125/MUC16 may lead to MMP-9 increased activity and invasiveness. Akt activation has been associated with the induction of EMT in carcinoma cells (Grille *et al*, 2003; Yan *et al*, 2009). These data are consistent with the observation that Akt is activated in knockdown cells. Our finding provides mechanistic support to a previous study, which showed that CA125/MUC16 tissue loss (extracellular region) is associated with poor prognosis in EOC (Hogdall *et al*, 2007).

In conclusion, we provide direct evidence that the knockdown of CA125/MUC16 in NIH:OVCAR3 ovarian cancer cells alters epithelial and mesenchymal markers, cell motility and migration. The underlying mechanism involves, at least in part, the activation of EGFR and its downstream signalling pathway.
